# RNAi Screen in *Drosophila* Cells Reveals the Involvement of the Tom Complex in *Chlamydia* Infection

**DOI:** 10.1371/journal.ppat.0030155

**Published:** 2007-10-26

**Authors:** Isabelle Derré, Marc Pypaert, Alice Dautry-Varsat, Hervé Agaisse

**Affiliations:** 1 Section of Microbial Pathogenesis, Yale University School of Medicine, New Haven Connecticut, United States of America; 2 Department of Cell Biology, Yale University School of Medicine, New Haven, Connecticut, United States of America; 3 Center for Cell and Molecular Imaging, Yale University School of Medicine, New Haven, Connecticut, United States of America; 4 Unité de Biologie des Interactions Cellulaires, Institut Pasteur, Paris, France; 5 CNRS URA 2582, Paris, France; Stanford University, United States of America

## Abstract

*Chlamydia* spp. are intracellular obligate bacterial pathogens that infect a wide range of host cells. Here, we show that C. caviae enters, replicates, and performs a complete developmental cycle in *Drosophila* SL2 cells. Using this model system, we have performed a genome-wide RNA interference screen and identified 54 factors that, when depleted, inhibit C. caviae infection. By testing the effect of each candidate's knock down on L. monocytogenes infection, we have identified 31 candidates presumably specific of *C. caviae* infection. We found factors expected to have an effect on *Chlamydia* infection, such as heparansulfate glycosaminoglycans and actin and microtubule remodeling factors. We also identified factors that were not previously described as involved in *Chlamydia* infection. For instance, we identified members of the Tim-Tom complex, a multiprotein complex involved in the recognition and import of nuclear-encoded proteins to the mitochondria, as required for C. caviae infection of *Drosophila* cells. Finally, we confirmed that depletion of either Tom40 or Tom22 also reduced C. caviae infection in mammalian cells. However, C. trachomatis infection was not affected, suggesting that the mechanism involved is C. caviae specific.

## Author Summary

Chlamydia spp. are intracellular bacterial pathogens that infect a wide range of hosts and cause various diseases, including preventable blindness in developing countries, sexually transmitted disease, and pneumonia. Chlamydia spp. are able to establish their replication niche inside the host cell, residing in a membrane-bound compartment that serves as a protector shield against immune surveillance and antimicrobial agents but also acts as a “filter” to exchange factors with the host cell. Despite the primary importance of *Chlamydia* for human health, little is known about the mechanisms underlying the infection process. The study of *Chlamydia* pathogenesis is challenging because *Chlamydia* spp*.* are not amenable to genetic manipulation and it is difficult to conduct extensive genetic approaches in the mammalian host. To circumvent these difficulties, we have used *Drosophila* cells to model *Chlamydia* infection. We conducted a genome-wide RNA interference screen and identified host factors that, when depleted, reduce *Chlamydia* infection. Validating our approach, we further showed that the identified factors were also required for infection in mammalian cells. This work will help us better understand the complex interaction between *Chlamydia* and its host and potentially identify novel targets for therapeutic treatment.

## Introduction

Chlamydia spp. are Gram-negative, obligate, intracellular bacterial pathogens that infect a wide range of hosts and cause various diseases. Three species infect humans. C. trachomatis is the leading cause of preventable blindness in developing countries [1] and the most common cause of bacterial sexually transmitted disease in developed countries [2]. Infection with C. pneumoniae leads to pneumonia, and in the past 10 years, C. pneumoniae has been implicated in atherosclerosis [3] and Alzheimer disease [4], although the direct links between the bacteria and these diseases is still unclear. C. psittaci infects various animals and is responsible for pneumonia in humans [5]. Many *Chlamydia* species are recognized as animal pathogens [6]. C. muridarum infects mice and hamsters. C. suis, C. abortus, and C. felis infect swine, ruminants, and house cats, respectively. Finally, infection with C. caviae in guinea pig resembles ocular and genital infections caused by C. trachomatis in humans.

*Chlamydia* are characterized by a biphasic developmental cycle that occurs exclusively in the host cell. The bacteria alternate between an infectious, metabolically inactive form called elementary body (EB) that is characterized by a condensed nucleoid, and an intracellular, metabolically active form named reticulate body (RB). Once internalized, *Chlamydia* resides in a membrane-bound compartment, named the inclusion. Shortly after uptake, an uncharacterized switch occurs, leading to the differentiation of EBs into RBs. The RBs then start to replicate until the inclusion occupies a large part of the cytosol of the host cells. At the end of the cycle, which lasts 2 to 3 d depending upon the species, the RBs differentiate back into EBs. The host cell is lysed, leading to the release of EBs and the infection of neighboring cells [7,8].

Both bacterial and host factors contribute to the biogenesis of the inclusion, but little is known about the mechanisms involved. Chlamydia spp. possess a type III secretion system (TTSS) responsible for the secretion of effector proteins in the cytoplasm of the host cell. An example of such effectors is the family of highly hydrophobic Inc proteins. Some of them are present on the surface of the inclusion membrane and are thought, in combination with other bacterial effector proteins, to modify the host cell environment and allow bacterial replication [9–13]. During the cycle, *Chlamydia* targets various host cell functions in order to establish its replication niche and disseminate from cell to cell [14]. The bacteria acquire amino acids, nucleotides, and other precursors from the host cell. The mechanism of chlamydial entry is not well understood, but among others, heparan sulfate proteoglycans, tyrosine phosphorylation of the bacterial effector Tarp, and activation of small GTPases and signaling pathways leading to actin remodeling are involved in this process [15]. Once internalized, *Chlamydia* directs the trafficking of the nascent inclusion to a perinuclear localization via a mechanism involving microfilaments, microtubules, and the motor protein dynein [16]. The inclusion does not interact with the endocytic pathway [14,17]. However, it intercepts exocytic vesicles and lipids from the Golgi [18]. Some Rab GTPases are recruited to the inclusion membrane [19], and a recent study suggests that *Chlamydia* targets host lipid droplets to enhance its intracellular survival and replication [20]. Finally, *Chlamydia* has the ability to modulate the programmed cell death pathway of infected cells [21,22]. During the early stage of infection, the infected cells are resistant to apoptosis signals but, by the end of *Chlamydia* developmental cycle, the programmed cell death pathway is induced, presumably to facilitate the release of the bacteria and the initiation of the next round of infection.

In the past few years, *Drosophila* has been established as a useful model to dissect microbial pathogenesis [23]. Among others, Pseudomonas aeroginosa [24], Mycobacterium marinum [25], *Salmonella* [26], and *Listeria monocytogenes* [27] successfully infect *Drosophila* adult flies. Host–pathogen interaction can also be analyzed in *Drosophila* S2 cells, which resemble embryonic hemocytes/macrophages. For example, the intracellular replication of L. monocytogenes [27,28] or Legionella pneumophila [29] in *Drosophila* cell lines is similar to the one observed in mammalian cells, and the first steps, but not the latest (RB to EB differentiation), of C. trachomatis developmental cycle can be observed in *Drosophila* cells [30].

An important discovery was made by Clemens et al., who reported that the simple addition of dsRNA to *Drosophila* cells in culture reduces or eliminates the expression of target genes by RNA interference (RNAi), thus efficiently phenocopying loss-of-function mutations [31]. Combined with the sequence of the *Drosophila* genome, it has opened a new area of research, allowing scientists to test the involvement of any *Drosophila* gene in a given cellular process [32,33]. Several screens have already shed light on various cellular processes such as cell viability [33], cytokinesis [34], wnt signaling [35], JAK/STAT signaling [36], and mechanisms of host–pathogen interaction, including *Listeria* and *Mycobacterium* pathogenesis [37–39], Candida albicans phagocytosis [40], and L. pneumophila exploitation of the early secretory pathway [29].

We have investigated the possibility of using *Drosophila* Schneider's Line 2 (SL2) cells [41] as a model system to dissect *Chlamydia* pathogenesis. We have shown that C. caviae enters, replicates, and performs a complete developmental cycle in *Drosophila* SL2 cells. We performed a genome-wide RNAi screen and identified 54 factors that, when depleted, inhibit C. caviae infection in *Drosophila* cells. We identified factors expected to have an effect on *Chlamydia* infection, but most importantly we also identified uncovered host factors, including components of the Tim-Tom complex. Clearly validating our approach, we showed that depletion of either Tom40 or Tom22 also reduced C. caviae infection in mammalian cells. We discuss how further investigation of the identified candidates may shed light on the molecular mechanisms involved in *Chlamydia* pathogenesis.

## Materials and Methods

### Cell Lines and Bacterial Strains

*Drosophila* SL2 cells [41] were cultured at 25 °C in Schneider media (Invitrogen) supplemented with 10% heat inactivated FBS (JRH).

HeLa 229 cells were cultured at 37 °C with 5% CO_2_ in DMEM high glucose (Invitrogen) supplemented with 10% heat inactivated FBS (Invitrogen).

C. caviae, the guinea pig model of genital and ocular infection of C. trachomatis, were obtained from R. Rank (University of Arkansas). *C. trachomatis Lymphogranuloma venerum, Type II*, were obtained from ATCC (VR-902B).

SL2 cell infection with GFP-expressing L. monocytogenes was conducted as previously described [37].

### *Chlamydia* Propagation and Infection

For propagation, HeLa 229 were incubated with C. caviae or C. trachomatis for 48 h in the presence of 2 μg/ml cycloheximide (Sigma). The infected cells were centrifuged (10 min, 1,000 rpm) and the cell pellet was resuspended in SPG buffer (218 mM sucrose, 3.76 mM KH_2_PO_4_, 7.1 mM KH_2_PO_4_, 4,9 mM glutamate [pH 7.4]). The cells were broken by passing them through a 26^1/2^ gauge needle and the unbroken cells and nuclei were pelleted by centrifugation (10 min, 1,000 rpm). The supernatant was centrifuged (30 min, 12,000 rpm), and the bacterial pellets were resuspended in SPG buffer and stored at −70 °C.

For *Drosophila* SL2 cell infection, C. caviae were diluted in Schneider media supplemented with 10% heat inactivated FBS and incubated with the cells at 30 °C for the indicated time. For HeLa 229 cell infection, C. caviae or C. trachomatis were diluted in DMEM high glucose supplemented with 10% heat inactivated FBS and incubated with the cells at 37 °C in the presence of 5% CO_2_. One hour post infection, the bacteria were washed away and the cells were incubated with fresh media for the indicated length of time at 37 °C in the presence of 5% CO_2_.

### Antibodies

The following primary antibodies were used: (FITC)-conjugated C5+C8 monoclonal antibodies directed against *Chlamydia* MOMP and LPS (1:300, Argene), rabbit polyclonal anti IncA (1:200, [42]), guinea pig polyclonal antibody directed against C. caviae EBs (Kind gift of R. Rank, University of Arkansas), rabbit polyclonal antibody anti-hTom40 (1:500, Kind gift of M. Ryan, La Trobe University, Australia [43]), mouse monoclonal anti-Tom22 (1:2000, Sigma, clone 1C9–2), and rabbit polyclonal anti-actin (1:10,000, Sigma A2066).

The following secondary antibodies were used: goat anti-rabbit AlexaFluor 594 antibody (1:1,000, Molecular Probes), fluorescein (FITC)-conjugated AffiniPure donkey anti-guinea pig IgG (1:500, Jackson ImmunoResearch), peroxidase-conjugated goat anti-rabbit IgG (1:10,000, Jackson ImmunoResearch), and peroxidase-conjugated goat anti-mouse IgG (1:10,000, Jackson ImmunoResearch).

### Immunofluorescence

At the indicated time, the cells were fixed for 30 min in PBS containing 4% paraformaldehyde. Immunostainings were performed at room temperature. Antibodies were diluted in PBS containing 0.16 μg/ml Hoechst (Molecular Probes), 0.1% BSA, and 0.05% saponin. Samples were washed with PBS containing 0.05% saponin, and a final PBS wash was performed before examination under an epifluorescence microscope.

### Electron Microscopy

*Drosophila* SL2 cells (10^8^) were incubated at 30 °C with C. caviae (MOI ∼ 5), fixed 45 h post infection by addition of 0.125% glutaraldehyde / 2% paraformaldehyde in 0.1 M phosphate buffer (pH 7.4), postfixed with osmium tetroxide, dehydrated in ethanol, embedded in epoxy resin, sectioned, stained with 1% uranyl acetate, and examined by electron microscopy [44].

HeLa 229 cells cultured on coverslips were fixed in 2.5% glutaraldehyde in 0.1 M sodium cacodylate (pH 7.4) for 1 h at room temperature, postfixed in 1% osmium tetroxide in the same buffer for 1 h at room temperature, stained in 2% uranyl acetate in 50 mM sodium maleate (pH 5.2) for 1 h at room temperature, dehydrated in ethanol, and embedded in Embed 812 epoxy resin (all reagents from Electron Microscopy Sciences). Ultra-thin sections (60 nm) were obtained on a Reichert ultra microtome, transferred onto formvar- and carbon-coated hexagonal nickel grids, stained with 1% lead citrate and 2% uranyl acetate, and examined in a Tecnai 12 Biotwin electron microscope (FEI Company). Random images of vacuoles were recorded at a magnification of 11,500 using a Morada CCD camera (Olympus Soft Imaging Solutions). For quantitation of the percentage of vacuolar membrane or nuclear envelope covered by mitochondria, a grid with a distance of 560 nm between lines was superposed on top of the images, and the number of intersections of vertical and horizontal lines with membranes counted. The number of intersections of these lines with mitochondria was also counted, but mitochondria were counted as being associated with the vacuolar or nuclear membrane only if the distance between the point of intersection of the grid with the mitochondrial outer membrane and the closest vacuolar or nuclear membrane was 50 nm or less. The ratio of the number of intersection with mitochondria divided by the number of intersections with the vacuolar or nuclear membrane gives an estimate of the percentage of these membranes covered by mitochondria.

### Infectious Progeny Production in *Drosophila* Cells

*Drosophila* SL2 cells (10^8^) were incubated at 30 °C with C. caviae. At the indicated time, the infected cells were processed as described above for *Chlamydia* propagation. The bacterial pellets were resuspended in 100 μl of SPG. To test for the presence of infectious C. caviae in the preparation, 300 μl of a 1:100 dilution were incubated with 6.10^4^ HeLa cells seeded onto coverslips at 37 °C in the presence of 5% CO_2_. After 1 h, the bacterial suspension was replaced by 500 μl of fresh medium. The cells were fixed 24 h post infection, stained, and the percentage of cells containing a large inclusion was determined by visual inspection using an epifluorescence microscope.

### Infectious Progeny Production in HeLa 229 Cells

The infection was performed in 384-well format such that 75% of the cells were infected. At the indicated time, the infected cells were collected and transferred to an eppendorf tube containing 100 μl of glass beads (Sigma, G8772) and 300 μl of DMEM high glucose supplemented with 10% FBS. The cells were broken by vortexing for 1 min, and 40 μl of dilutions of the lysat were added to 4.10^3^ HeLa 229 cells seeded in 384-well plate. After 1 h at 37 °C in the presence of 5% CO_2_, the lysat was washed away and 40 μl of fresh media was added to each well. The cells were fixed and stained 24 h post infection and the percentage of infected cells was determined.

### Primary Screen

Two sets of 42 384-well plates containing 0.25 μg of dsRNA per well were provided by the *Drosophila* RNAi Screening Center (Harvard Medical School, Boston, Massachusetts, http://www.flyrnai.org). *Drosophila* SL2 cells (2.10^4^), resuspended in 20 μl of serum-free Schneider media, were seeded in each well and incubated 1 h at 25 °C before the addition of 20 μl of Schneider media containing serum. After 3.5 d, the cells were infected by addition of 10 μl of Schneider media containing C. caviae. The cells were centrifuged for 1 min at 1,000 rpm and incubated at 30 °C for 48 h. The cells were processed for immunofluorescence by using the DNA dye Hoeschst and FITC-conjugated C5+C8 monoclonal antibodies. An automated microscope was used to automatically track, focus, and capture fluorescent images of the cells within each well across an entire plate. One set of images was captured in the blue channel to detect the cells' nuclei and one set in the green channel to detect *Chlamydia*. The qualitative analysis of the image data was done by visual inspection.

### dsRNA Synthesis

dsRNA used for validation and secondary assays were synthesized using a MEGAscript High Yield transcription kit (Ambion) according to the recommendation of the manufacturer.

### Tom40 and Tom22 siRNA in HeLa 229 Cells

The protocol used for siRNA transfection was adapted from Dharmacon's HeLa cells transfection protocol. One volume of siRNA buffer containing 200 nM of siRNA was incubated with 1 volume of serum-free DMEM high glucose containing 5 μl/ml DharmaFECT-1 transfection reagent for 20 min at room temperature. Two volumes of DMEM high glucose supplemented with 20% FBS containing 5.10^4^/ml HeLa 229 cells were added to each well and the cells were incubated at 37 °C with 5% CO_2_ for 3 d. The total volume was 40 μl in 384-well and 400 μl in 24-well. In 24-well format the transfection mix was replaced by 500 μl of fresh media 24 h post transfection.

### Assay for Tom40 and Tom22 Protein Depletion

The knock down of Tom40 or Tom22 was performed as described above in 24-well plate. Three days post transfection, the cells were harvested in 100 μl of protein sample buffer and 20 μl of cell lysates were run on SDS-PAGE gels and analyzed by western blot using HPR-conjugated secondary antibodies and Amersham ECL western blotting detection reagents.

### Computer Assisted Image Analysis

Images were acquired using the Metamorph software (Molecular Devices). The integrated morphometry analysis module was used to quantify the size of C. caviae inclusions.

## Results

### C. caviae Infect and Replicate in *Drosophila* SL2 Cells

In an attempt to use *Drosophila* as a model system to study *Chlamydia* pathogenesis, we investigated C. caviae replication in *Drosophila* SL2 cells. For this purpose, 80% confluent *Drosophila* SL2 cells cultured in 96-well dish were incubated with C. caviae. At various times post infection, the cells were transferred to Concanavalin A–coated coverslips (Sigma, 2 mg/ml) in Schneider media for 2 h. The samples were then fixed and stained with the DNA dye Hoescht and a FITC-conjugated antibody directed against *Chlamydia* to stain the inclusion.

**Figure 1:**
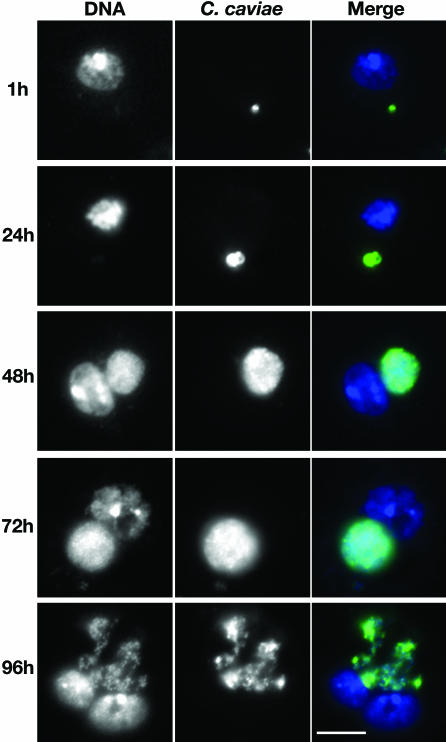
C. caviae Replicate in *Drosophila* SL2 Cells Immunofluorescence images of *Drosophila* SL2 cells incubated for 1, 24, 48, 72, and 96 h with *C. caviae*. After fixation, the samples were stained with the DNA dye Hoechst (DNA, blue) and a FITC-conjugated monoclonal C5+C8 antibody directed against MOMP and LPS (C. caviae, green). Merge: overlay of the two images.

As shown in Figure 1, C. caviae is able to infect and replicate in *Drosophila* SL2 cells. Although most of the cells contained at least one bacterium 1 h post infection, only 20% to 30% of the cells had an inclusion 48 h post infection (not shown), suggesting that some bacteria were actually cleared in the phagocytic SL2 cells. However, when the bacteria were successful in establishing their niche, the infected cells displayed a perinuclear inclusion whose size increased between 24 and 72 h post infection. At 96 h post infection, the size of the inclusions was more heterogeneous (not shown) and some cells displayed disrupted inclusions, suggesting that the developmental cycle was completed and that reinfection was occurring between 72 and 96 h post infection.

### C. caviae Complete a Full Developmental Cycle in *Drosophila* SL2 Cells

**Figure 2:**
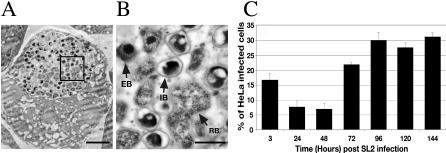
C. caviae Undergo a Full Developmental Cycle in *Drosophila* SL2 Cells (A) Electron micrograph of semi-thin sections (∼70 nm) of *Drosophila* SL2 cells 45 h post infection with C. caviae. Black square: Area magnified and shown in (B). Bar: 2 μm. (B) Higher magnification of the bacteria of the C. caviae inclusion shown in (A). Bar: 500 nm. (C) C. caviae were isolated from *Drosophila* SL2 cells 3, 24, 48, 72, 96, 120, and 144 h post infection and were used to infect HeLa cells for 24 h. The percentage of HeLa cells that present an inclusion is shown.

We next determined whether C. caviae were undergoing a full developmental cycle in *Drosophila* SL2 cells. To this end, we determined whether the different developmental forms of C. caviae were present in the inclusion by electron microscopy. As shown in Figure 2A, 45 h post infection the bacteria were found in a membrane-bound compartment that occupies most of the cytosolic space. The inclusions mainly contained RBs and intermediate bodies (IBs) in the process of differentiating to EBs and are characterized by their DNA condensation stage, but they also contained some bacteria with an EB morphology (Figure 2B), suggesting that in *Drosophila* SL2 cells, RBs start to differentiate back to EBs 45 h post infection.

In order to demonstrate that infectious progeny was produced, C. caviae harvested from *Drosophila* SL2 cells at different times post infection were used to infect HeLa cells (Materials and Methods; Figure 2C). When C. caviae were harvested 3 h post SL2 infection, 10% of the HeLa cells displayed an inclusion. This number decreased to less than 5% when the bacteria were isolated 24 or 48 h post infection, suggesting that a substantial amount of bacteria were either cleared or had differentiated into non-infectious RBs. In contrast, 12.5% and 19% of the HeLa cells contained a large inclusion when the bacteria were harvested 72 and 96 h post SL2 infection, respectively. After 96 h, the number of infected HeLa cells remained constant. These results indicate that infectious forms of C. caviae are produced in *Drosophila* SL2 cells. Moreover, they are in agreement with the immunofluorescence (Figure 1) and electron microscopy (Figure 2A and 2B) data and confirm that 48 h post infection of *Drosophila* SL2 cells, the inclusion mainly contains RBs and IBs, whereas EBs are produced in the next 24 h. Taken together, these data show that C. caviae undergo a full developmental cycle in *Drosophila* SL2 cells and suggest that the cycle lasts 72 to 96 h.

### C. caviae TTSS Is Functional in *Drosophila* SL2 Cells

**Figure 3:**
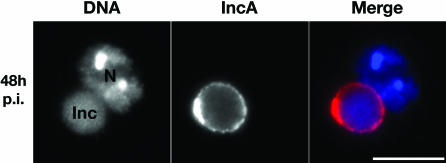
C. caviae TTSS Is Functional in *Drosophila* SL2 Cells *Drosophila* SL2 cells infected for 48 h with C. caviae, were stained with the DNA dye Hoechst (DNA, blue) and polyclonal antibodies directed against the IncA proteins (IncA, red). Merge: overlay of the two images. N: nucleus; Inc: inclusion. Bars: 10 μm.

The TTSS of C. caviae was functional in *Drosophila* SL2 cells as shown by determining the presence of the Inc family protein, IncA, on the C. caviae inclusion membrane (Figure 3). *Drosophila* SL2 cells were fixed 48 h post infection with C. caviae, stained with the DNA dye Hoescht to visualize the nuclei (N) and the inclusions (Inc), and antibodies directed against IncA. A ring-like signal (IncA, red) that surrounded the inclusion (Inc, blue) was observed, indicating that, in *Drosophila* SL2 cells, the TTSS of C. caviae is functional and that TTS substrates such as IncA, are delivered to the inclusion membrane.

### Host Factors That, When Depleted, Inhibit C. caviae Infection

**Figure 4:**
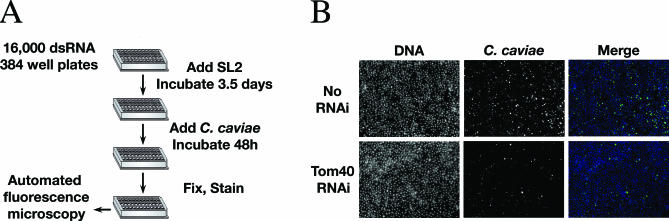
Genome-Wide RNAi Screen to Identify Host Factors Involved in C. caviae Infection (A) Schematic representation of the screening procedure. dsRNA were incubated with *Drosophila* SL2 cells for 3.5 d before incubation with C. caviae for 48 h. After fixation and staining, images were acquired using an automated microscope. (B) Illustration of C. caviae growth inhibition phenotype that was selected in the primary screen for further analysis. *Drosophila* SL2 cells were incubated with Tom40 dsRNA (Tom40 RNAi) or with buffer alone (No RNAi) for 3.5 d and were subsequently incubated with C. caviae for 48 h. The samples were fixed and stained with the DNA dye Hoechst (DNA, blue) and a FITC-conjugated monoclonal C5+C8 antibody directed against MOMP and LPS (C. caviae, green). Merge: overlay of the two images.

**Figure 5:**
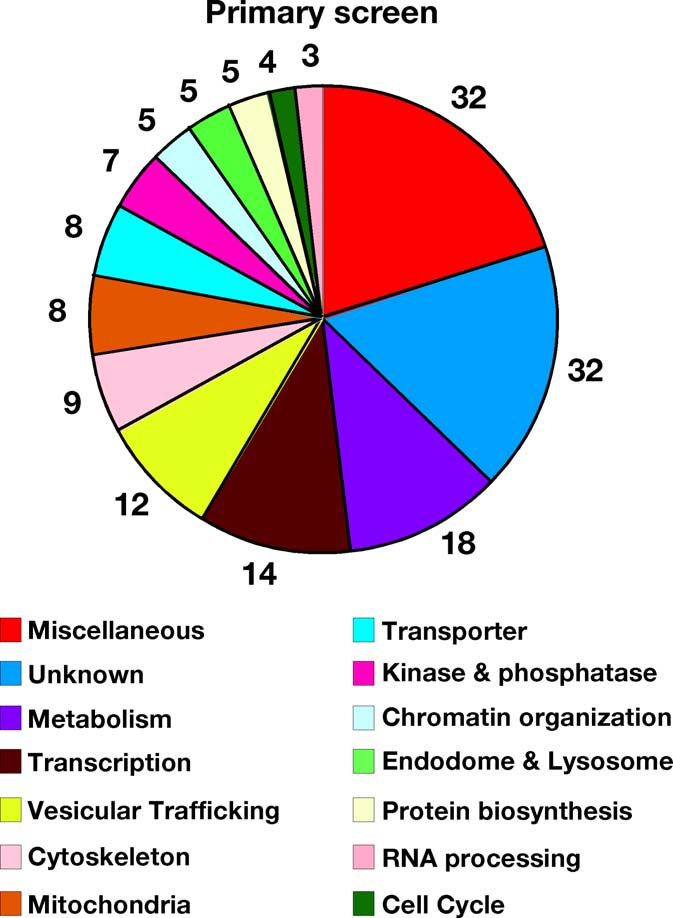
Functional Categories of the 162 Candidates Identified in the Primary Screen Based on their predicted biological functions, the 162 candidates identified in the primary screen were categorized in 14 functional groups. The number surrounding the chart indicates the number of candidates in each category. See Table S1 for the list of candidates.

Sixteen thousand *Drosophila* genes were individually knocked down by RNAi and screened for their ability to reduce C. caviae infection of *Drosophila* SL2 cells. The assay was performed as follows (Materials and Methods; Figure 4A). After 3.5 d of RNAi treatment, the *Drosophila* SL2 cells were incubated with C. caviae for 48 h. The infected cells were fixed and stained with a DNA dye and a *Chlamydia*-specific FITC-conjugated antibody. An automated microscope was used to capture fluorescence images that were subsequently analyzed by visual inspection. The primary screen was performed in duplicate. We identified 162 candidates that, when depleted, reduced C. caviae infection (Table S1). Figure 4B is representative of the phenotype observed: few cells displayed wild-type size inclusion (middle top panel) and the number of infected cells, as well as the size of the inclusion, was largely reduced (middle bottom panel). The candidates were grouped into 14 functional categories (Figure 5): miscellaneous (32), unknown (32), metabolism (18), transcription (14), vesicular trafficking (12), cytoskeleton (9), mitochondria (8), transporter (8), kinase and phosphatase (7), chromatin organization (5), endosome and lysosome (5), protein biosynthesis (5), RNA processing (4), and cell cycle (3).

The dsRNA targeting most of the candidates of the miscellaneous, metabolism, vesicular trafficking, cytoskeleton, mitochondria, transporter, kinase and phosphatase, and endosome and lysosome categories were resynthesized to confirm the phenotype observed in the primary screen (Table S1). Out of the 100 candidates retested, the phenotype was confirmed for 54 candidates in at least two out of three replicates (Table 1). The validation rate varied among the categories: miscellaneous (40%), metabolism (47%), vesicular trafficking (75%), cytoskeleton (75%), mitochondria (67%), transporter (37%), kinase and phosphatase (57%), and endosome and lysosome (100%).

**Table 1:**
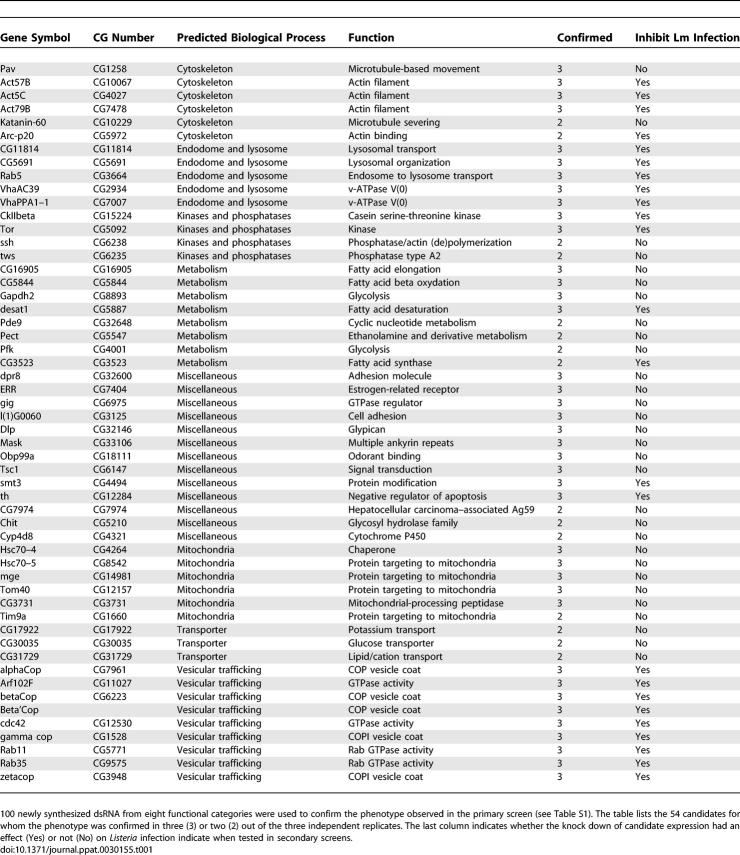
Host Factors Required for Optimal C. caviae Infection and Their Effect on L. monocytogenes Infection

In an attempt to assay for *Chlamydia* specificity, the knock down of the candidates was tested for the inhibition of L. monocytogenes infection (Table 1). The knock down of most vesicular trafficking (9/9), cytoskeleton (4/6), and endosome and lysosome (5/5) candidates inhibited both C. caviae and L. monocytogenes infection, and an equal number of kinase and phosphatase candidates inhibited C. caviae or L. monocytogenes infection. The knock down of most miscellaneous (11/13) and metabolism (6/8) candidates and of all mitochondria (6/6) and transporter (3/3) candidates inhibited C. caviae infection only. These results suggest that the latter categories are likely to represent candidates specifically involved in C. caviae infection.

### Tom40 and Tom22 Depletion Reduce C. caviae Infection in Mammalian Cells

The RNAi screen in *Drosophila* cells revealed that the silencing of six mitochondrial genes inhibited C. caviae, but not L. monocytogenes infection. Moreover, four out of the six candidates were members of the mitochondrial membrane translocase, a multiprotein complex involved in the recognition and import of nuclear-encoded mitochondrial proteins to the mitochondria [43,45]. Taken together, these results suggested a specific role of this machinery for optimal C. caviae infection in *Drosophila* cells. To address the relevance of these findings in *Chlamydia* pathogenesis, this observation was further investigated in mammalian cells.

**Figure 6:**
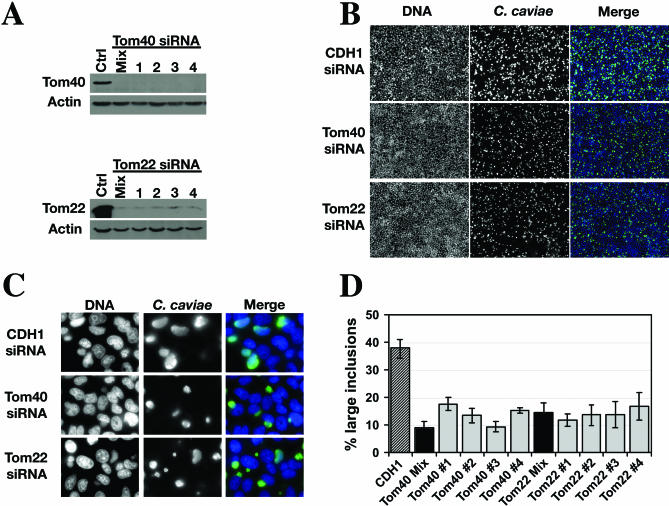
Tom40 and Tom22 Depletion Reduce C. caviae Infection of Mammalian Cells (A) HeLa 229 cells were transfected for 3 d with a CDH1 control siRNA (Ctrl), or four siRNA duplexes against Tom40 or Tom22 either pooled (Mix) or individually (1, 2, 3, 4) and analyzed by immunoblotting using antibodies directed against Tom40, Tom22, or actin. (B and C) Immunofluorescence images of HeLa 229 cells transfected for 3 d with CDH1, Tom40, or Tom22 siRNA and subsequently incubated for 24 h with C. caviae. After fixation the cells were stained with the DNA dye Hoechst (DNA, blue) and polyclonal antibodies against C. caviae (C. caviae, green). Merge: overlay of the two images. (B) Low magnification images (10×). (C) High magnification images (100×). (D) Quantification of the percentage of large C. caviae inclusions upon CDH1, Tom40, or Tom22 siRNA. Inclusions were defined as objects whose size ranged from 10 to 150 μm^2^. Objects whose size ranged from 30 to 150 μm^2^ were defined as large inclusions. Mix: pool of four siRNAs; 1, 2, 3, 4: individual siRNA.

Tom40 or Tom22 expression was knocked down in HeLa 229 cells using a mix of four siRNA duplexes directed against their respective mRNA (ThermoFisher). In addition, each siRNA was tested individually to rule out any potential off-target effects. The depletion of either Tom40 or Tom22 was assayed 3 d post transfection of the siRNAs by western blot analysis. As shown in Figure 6A, both Tom40 and Tom22 were efficiently depleted after incubation with the mix of four siRNAs or with individual siRNA duplexes.

The effect of Tom40 or Tom22 depletion on C. caviae infection was analyzed. HeLa 229 cells were incubated for 3 d with either CDH1 siRNA control directed against E-Cadherin, or Tom40 or Tom22 siRNAs pooled (mix), or individually (1, 2, 3, 4), infected with C. caviae for 24 h, and processed for immunofluorescence. The corresponding low and high magnification images are depicted in Figure 6B and 6C, respectively. The nuclei were labeled with the DNA dye Hoeschst (Figure 6B and 6C: left panel, DNA, blue) and the inclusions were stained with a guinea pig polyclonal antibody against C. caviae (Figure 6B and 6C: middle panels, C. caviae, green). Although the number of infected cells was similar, the inclusions appeared smaller upon Tom40 or Tom22 depletion (compare CDH1 middle panels to Tom40 or Tom22 middle panels).

A computer-assisted analysis of the images was used to quantify the size of the inclusions (Materials and Methods). In the control situation, we determined that each inclusion could be defined as a 10- to 150-μm^2^ object and 40% of the inclusions were larger than 30 μm^2^. We defined 10- to 30-μm^2^ and 30- to 150-μm^2^ objects as small and large inclusions, respectively. The impact of Tom40 or Tom22 knock down on C. caviae ability to form large inclusions was analyzed (Figure 6D). A 5- to 3-fold reduction in the percentage of large inclusions was observed upon depletion of either Tom40 or Tom22, confirming the overall reduction in the size of the inclusions and suggesting that upon Tom40 or Tom22 depletion, C. caviae intracellular growth is impaired.

**Figure 7:**
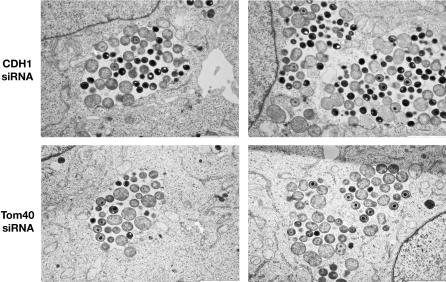
Tom40 Depletion Affects C. caviae Inclusion Size and Differentiation into EBs Electron micrographs of HeLa229 cells depleted for CDH1 (top panels, CDH1 siRNA) or Tom40 (bottom panels, Tom40 siRNA) 24 h post C. caviae infection. Representative images of small (left panels) and large (right panels) inclusions are shown. Bar: 2 μM.

Electron microscopy analysis of C. caviae inclusions in control or Tom40 depleted cells confirmed the immunofluorescence results. Although a mixed population of small and large inclusions was observed 24 h post infection, the overall size of Tom40 depleted cell inclusion was smaller (Figure 7). In addition, RBs had already started to differentiate back into EBs in control cells, and 85% of the inclusions contained more than 25% EBs. In contrast, although some IBs were present, very few EBs were visible in Tom40-depleted cells, and only 25% of the inclusions contained more than 25% EBs. This result suggested that, in addition to a reduction in intracellular growth, differentiation back into EBs is also lessened in Tom40-depleted cells.

### C. caviae, but Not C. trachomatis, Infectious Progeny Production Is Impaired upon Tom40 and Tom22 Depletion in Mammalian Cells

**Figure 8:**
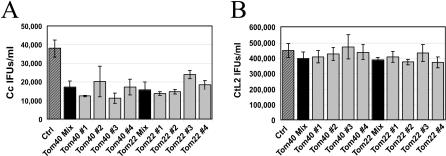
Tom40 and Tom22 Depletion Reduces the Production of C. caviae, but Not C. trachomatis, Infectious Progeny Quantification of the infectivity of C. caviae (A) or C. trachomatis (B) progeny isolated from CDH1 or Tom40- or Tom22-depleted cells. The progeny was isolated 48 h post infection and dilutions were incubated with fresh HeLa 229 cells for 24 h. After fixation and staining, the IFUs/ml was determined by assessment of the number of infected cells. Cc: C. caviae; CtL2: C. trachomatis.

The electron microscopy results suggested that RB differentiation into EBs was reduced upon Tom40 or Tom22 depletion. We therefore investigated the production of infectious progeny by Tom40- or Tom22-depleted cells. The cells were incubated with the siRNA in pool or individually for 3 d before incubation with C. caviae for 48 h to allow completion of the developmental cycle. The infected cells were collected, lysed with glass beads, and dilutions of the lysate were used to infect fresh HeLa 229 cells (see Materials and Methods). The cells were fixed 24 h post infection and the number of inclusion forming units (IFUs) was determined after assessment of the number of infected cells by immonulabeling (Figure 8A). We observed a 2- to 3-fold reduction in the production of infectious progeny upon Tom40 or Tom22 depletion. On the contrary, a similar number of infectious C. trachomatis were recovered from control or Tom40- or Tom22-depleted cells (Figure 8B).

These results demonstrate that, as suggested by the electron microscopy analysis, the reduction in the size of C. caviae inclusions is accompanied with a decrease in the number of infectious progeny produced. Altogether, our results indicate that depletion of members of the Tom complex in mammalian cells have a detrimental effect on C. caviae intracellular replication, which impairs bacterial replication and differentiation. Since Tom40 or Tom22 depletion had no effect on C. trachomatis infection, our results also indicate that the mechanism involved is C. caviae–specific.

## Discussion

*Chlamydia* infections represent an enormous burden to human health, and although some of the host cellular processes targeted by *Chlamydia* have been identified, most of the factors involved in the infection process remained to be identified. This paucity of knowledge is mainly due to the fact that *Chlamydia* is not genetically tractable and to the difficulty of conducting genetic approaches in the mammalian host. *Drosophila* has recently emerged as a powerful alternative model to dissect microbial pathogenesis, and we show here that *Drosophila* SL2 cells constitute a viable model to study *Chlamydia* infection and identify host factors involved in the infection process.

### C. caviae Replication in *Drosophila* Cells

We demonstrated that, similar to the situation in mammalian cells [8], infectious forms (EB) of C. caviae enter *Drosophila* SL2 cells, differentiate into the replicative form (RB), replicate within a membrane-bound compartment, and differentiate back from RBs to EBs.

A previous report showed that different serovars of C. trachomatis, including C. trachomatis LGV serovar L2, could initiate their developmental cycle in *Drosophila* S2 cells [30]. However, the later stages of the developmental cycle were not achieved. Similarly, we found that when *Drosophila* SL2 cells were incubated with C. trachomatis LGV serovar L2 most cells were also infected 1 h post infection. However, the pattern of staining did not change over a 72-h period post infection and the cells never displayed large perinuclear inclusions (not shown), confirming that C. trachomatis developmental cycle is not complete in *Drosophila* SL2 cells.

We noticed a difference in the morphology of C. caviae inclusion in *Drosophila* cells compared to mammalian cells. The inclusions appear multilobed in mammalian cells [46], whereas they appeared as a single membrane-bound compartment in *Drosophila* SL2 cells. This morphology resembles that of C. trachomatis inclusions that are known to undergo homotypic fusion and are therefore monovacuolar. This observation suggests that C. caviae inclusions may also undergo homotypic fusion in *Drosophila* SL2 cells.

IncA, a type III secretion (TSS) substrate known to be in involved in the homotypic fusion of the C. trachomatis inclusions [47], was present on the surface of C. caviae inclusion in *Drosophila* SL2 cells. Since IncA from C. caviae can interact with itself [48], it is possible that in *Drosophila* SL2 cells it participates to the biogenesis of a single large inclusion. If it is the case, some *Drosophila* factors probably interact with IncA and help promote the fusion. However, one cannot exclude that in *Drosophila* cells the homotypic fusion of C. caviae inclusions is IncA independent.

### Host Factors Identified in RNAi Screen

Using the *Drosophila* cell / C. caviae model system, we have performed an RNAi screen and identified 54 host factors that, when depleted, reduced C. caviae infection. By testing the effect of the candidates' knock down on L. monocytogenes infection, we have identified candidates presumably specific of *C. caviae* infection. In the following section, we discuss their potential relevance in *Chlamydia* pathogenesis.

The attachment of most *Chlamydia* species to the host cell is dependent on host cell heparan sulfate glycosaminoglycans (GAGs) [15]. C. caviae is no exception, because its adhesion is GAG dependent and can be blocked by heparin [49]. *Drosophila* contains two main glypicans: Dally (Division abnormally delayed) [50] and Dlp (Dally-like protein) [51,52]. They are composed of cell-surface heparan sulfate proteoglycans linked to the plasma membrane by a glycosyl phosphatidylinositol linker. Our screen showed that the knock down of *Dlp* reduced C. caviae infection, suggesting that Dlp may promote the attachment of C. caviae to the cell surface.

Activation of Rho family of GTPases and actin remodeling has also been implicated in *Chlamydia* entry [15]. For example, Cdc42 and actin polymerization are involved in C. caviae entry of mammalian cells [53], and we show here that their depletion also reduced infection of *Drosophila* cells. Rac1, which is also involved in C. caviae entry in mammalian cells [53], was not identified in our screen. The *Drosophila* genome contains two *rac* genes, and it is possible that the single knock down of one or the other was not sufficient to block C. caviae entry. In addition, we also identified Ssh, a phosphatase that controls actin reorganization through the dephosphorylation of cofilin [54]. Ssh was not previously reported to play a role in *Chlamydia* pathogenesis, but our data suggest that it may be implicated in regulating actin dynamics upon entry of *C. caviae.*

After their internalization, C. trachomatis EBs direct the nascent inclusion to a peri nuclear area. This movement is dependent of microtubules and the motor dynein [16].We have identified two candidates that are linked to motors and microtubules. The first candidate, *pav,* encodes a kinesin-like protein [55]. Although kinesin is not involved in the trafficking of C. trachomatis inclusion to the peri nuclear area [16], the microinjection of antibodies against kinesin prevents the recruitment of mitochondria to C. psittaci inclusions and delays the developmental cycle [56]. A defect in mitochondria recruitment to the inclusion may therefore explain the phenotype observed upon *pav* knock down in *Drosophila* cells. The potential importance of mitochondria in *Chlamydia* infection will be further discussed in the following section. The second candidate related to microtubule is katanin-60. In mammalian cells, katanin concentrates at the centrosome of the cell, where the p60 subunit exerts its microtubule severing activity and induces the release of microtubules from the centrosome [57]. C. trachomatis inclusions associate with centrosomes [58]. It is possible that *Chlamydia* interacts with the centrosome and induces a katanin-mediated local destabilization of the microtubule network, thus allowing the expansion of the inclusion.

A recent study revealed a dynamic interaction between multi-vesicular body–derived constituents and C. trachomatis inclusion [59]. We have identified two candidates involved in lysosomal transport (CG11814) and organization (CG5691), as well as two subunits of the v-ATPase (VhaAC39 and VhaPPA1–1). The identification of such factors suggests that, at some point during the developmental cycle, *Chlamydia* inclusions may interact with compartments of the endocytic pathway. Further analysis of theses candidates may shed light on the mechanism involved.

C. trachomatis inclusion also intercepts vesicles and lipids from the Golgi [18] and targets lipid droplets [20]. We have identified several enzymes involved in fatty acid synthesis, desaturation, elongation, and oxidation. The identification of such enzymes reinforces the idea that the acquisition of lipids is an important aspect of *Chlamydia* intracellular replication, and further investigation may shed light on the host metabolism pathways targeted by *Chlamydia*.

### *Chlamydia* and the Tom Complex

Our screen revealed that the knock down of members of the Tim-Tom complex, the multiprotein complex involved in the recognition and import of nuclear-encoded mitochondrial proteins to the mitochondria [45,60], inhibited C. caviae infection in *Drosophila* cells. Importantly, we have shown that the knock down of two major components of the outer membrane complex of mitochondria, Tom40 and Tom22, also inhibited C. caviae infection in mammalian cells. In the following section we discuss potential mechanisms that may explain the phenotype observed.

#### Metabolism.

Mitochondria are involved in carbohydrate and lipid metabolism. Depletion of Tom complex components in mitochondria may lead to the reduction of the intracellular ATP pool and interfere with C. caviae infection. Although *Chlamydia* species have the capacity to produce their own ATP [61], they also encode ATP transporters [62–64], suggesting that *Chlamydia* may utilize host cell ATP. In agreement with this assumption, it has been reported that *Chlamydia* infection leads to an increase in ATP production [65]. Using a Luminescent Cell Viability Assay (Promega), we have shown that the intracellular ATP levels were comparable in control, Tom40-, or Tom22-depleted cells (between 1,500 and 2,000 luminescence arbitrary units / 384 well) (not shown). This result suggests that production of energy is not dramatically affected upon depletion of Tom40 or Tom22. Moreover, addition of extra glucose up to 20 g/l, to increase the source of energy available, did not rescue the phenotype (not shown). Finally, *Listeria* infection, which requires ATP for the bacteria to polymerize actin and spread from cell to cell, was not affected (not shown), but most importantly, a similar amount of C. trachomatis infectious progeny was recovered from control or Tom40- or Tom22-depleted cells, suggesting that the C. trachomatis developmental cycle was not affected (Figure 8B). Taken together, these results suggest that energy depletion does not account for the phenotype observed.

#### Apoptosis.

Mitochondria play an important role in the control of apoptotic events, and *Chlamydia* inhibits apoptosis of the host cell by a mechanism that prevents mitochondrial release of cytochrome c [21,22]. The depletion of members of the Tom complex could interfere with this process and therefore affect C. caviae replication. However, Tom40- or Tom22-depleted cells infected with C. caviae did not display the characteristic condensed nuclear morphology of apoptotic cells (Figures 6B, 6C, and 7). This result suggests that premature cell death of the infected cells is not responsible for the inhibition of C. caviae infection.

#### Mitochondria recruitment to the inclusion.

It was previously shown that mitochondria are associated with C. caviae inclusions but not C. trachomatis inclusions [66,67]. We therefore investigated whether mitochondria were still recruited to the C. caviae inclusions in Tom40-depleted cells (Figure S1). Our results indicate that mitochondria were found in vicinity (<50 nM) of 8.5% of C. caviae inclusion membrane in control cells. Consistent with the specific recruitment of mitochondria to the inclusion membrane, only 1% of the nuclear membrane was covered with mitochondria. In Tom40-depleted cells, mitochondria were found in the vicinity of 5.8% of C. caviae inclusion membrane. Further experiments will be required to determine whether this slight reduction in mitochondria recruitment may account for the inhibition of C. caviae infection in Tom40-depleted cells.

#### Import of bacterial effectors.

Finally, it was previously shown that two enteropathogenic E. coli type III effectors, EspF and Map, are recognized and imported to the mitochondria through the Tim-Tom complex [68,69]. It is therefore possible that similar C. caviae type III effectors are targeted to the mitochondria via the Tom complex. Depletion of Tom complex components may therefore interfere with C. caviae ability to specifically modulate mitochondrial functions and impair the intracellular replication of the bacteria. Identification of such effectors, possibly through their mitochondria targeting sequence, and their further characterization might shed light on the significance of the association *C. caviae* inclusion with the mitochondria.

In conclusion, we have used *Drosophila* as a model system to identify host factors important in C. caviae replication. By comparative analysis, we have identified factors that are probably specific to *Chlamydia* pathogenesis and we validated the effect of two candidates (Tom40 and Tom22) in mammalian cells. Our results indicate that upon Tom 40 or Tom22 depletion, C. caviae replication and differentiation is lessened, whereas C. trachomatis infection is not affected. Further investigations are under way to elucidate the specific role of the Tom complex in C. caviae infection.

## Supporting Information

Figure S1 Mitochondria Recruitment to C. caviae Inclusion (A) Electron micrographs of HeLa229 cells depleted for CDH1 (left panels, CDH1 siRNA) or Tom40 (right panels, Tom40 siRNA) 24 h post C. caviae infection. Representative images of mitochondria apposed to the inclusions membrane are shown. M: mitochondria. (B) Quantification of the fraction of inclusion or nuclear membrane covered with mitochondria (See Materials and Methods).
(4.8 MB TIF)

Table S1 Host Factors Required for Optimal C. caviae Infection Identified in the Primary Screen The gene name and the corresponding CG number of each candidates identified in the primary screen are shown. The functional categories were assigned based on gene ontology (GO) biological function terms and the annotation is based on GO molecular function, cellular component, or protein domains as indicated in FlyBase (http://www.flybase.org/).
(52 KB XLS)

### Accession Numbers

The National Center for Biotechnology Information (http://www.ncbi.nlm.nih.gov/) accession numbers for the mammalian genes are CDH1 (NM_004360), Tom40 (NM_006114), and Tom22 (NM_020243).

## Abbreviations

EB: elementary body
GAG: glycosaminoglycan
IB: intermediate body
RB: reticulate body
RNAi: RNA interference
SL2: Schneider's Line 2
TTSS: type III secretion system
